# A Friction-Driven Strategy for Agile Steering Wheel Manipulation by Humanoid Robots

**DOI:** 10.34133/cbsystems.0064

**Published:** 2023-11-20

**Authors:** Zhaoyang Cai, Xin Zhu, Pierre Gergondet, Xuechao Chen, Zhangguo Yu

**Affiliations:** ^1^ School of Mechatronical Engineering, Beijing Institute of Technology, Beijing, China.; ^2^ CNRS-AIST Joint Robotics Laboratory, IRL, Tsukuba, Japan.

## Abstract

Vehicle driving can substantially enhance the maneuverability of humanoid robots. Agile steering wheel manipulation requires rapid rotation in narrow spaces such as a cab, serving as the foundation for increasing driving speed, especially in an obstacle avoidance scenario. Generally, there are 3 human driving strategies, “Hand-to-Hand,” “Hand-over-Hand,” and “One-Hand.” Based on the human driving motion data, we quantitatively analyze these strategies from 3 aspects, motion range of joint combination, motion region of the shoulder, and velocity of the manipulation. Then, a friction-driven manipulation strategy using one hand is proposed utilizing the similarity between a humanoid robot and a driver (human). It effectively addresses the requirements of both a small range of motion and rapid manipulation. To prevent the deformation of the steering wheel caused by excessive force, we construct an operating force model specifically for the steering wheel. This model accurately describes the relationship between the rotation resistance and the state of the steering wheel. In addition, we propose a quadratic programming (QP)-based control framework to servo the robot to track the end-effector position and target wrench output by this model. Finally, the effectiveness of this paper is evaluated through an obstacle avoidance scenario, achieving a maximum rotation velocity of 3.14 rad/s.

## Introduction

Humanoid robots can potentially be used to perform tasks in hazardous situations, as they possess the ability to operate machinery designed for human use without the need for modification due to their human-like structure [1–3]. By integrating with a vehicle, robots can take advantage of the vehicle’s speed and agility to extend their working range. A humanoid robot can handle vehicles designed for humans and perform tasks such as turning valves or using tools on the vehicle in disaster response scenarios. Additionally, humanoid robots can accurately imitate human active protective motions during crashes, making them valuable for simulating real crash scenarios in car manufacturing. There have been some studies on humanoid robots driving vehicles [4].

In such studies, methods for mounting mechanical devices to steering wheels to enable robots to drive vehicles more easily have been proposed. A device for stable grasping is installed on the steering wheel, and one wrist joint is used for control, as proposed by HUBO Team [5]. In [6], the steering wheel is fitted with 2 parallel wooden poles. By placing a fully open clip between the poles, the Momaro robot can turn the steering wheel without gripping. A wooden bar is mounted on the steering wheel for humanoid robot driving [7]. Team IHMC mounts a unique mechanism on the steering wheel, such as a steering wheel assistive power booster for humans, and a robot can turn the wheel using its right arm [8]. Additionally, a handle is designed and fixed to the wheel to facilitate full and stable grasping, aligning the wrist axis with the steer axis [9]. In the aforementioned methods, some devices were installed on the steering wheels. These devices are adapted to the robot end-effectors, which allow the robot to steadily and quickly make turns. However, these devices that are mounted temporarily on vehicles by humans through designing and machining can lead to inefficiencies that are not ideal for tasks in risky situations.

Without the extra mechanical structure for the vehicle, several researchers have proposed using rods on end-effectors to turn the spokes of the steering wheel. One such method involves using a pair of metal rods on the left wrist that remain in firm contact with the steering wheel for turning purposes [10]. Another method involves using a peg on the hand to turn the steering wheel [11,12]. This approach allows the robot to move the steering wheel in a circle at a speed of up to 120^∘^/s. These methods use metal rods inserted between the spokes to quickly and steadily turn the wheel in one direction. However, when changing the steering direction, there is a gap that the peg must cross before contacting another spoke, during which the steering wheel is not in contact with the end-effector and is therefore in a state of loss of control and unable to change direction agility. In addition, these methods remove the A-pillar to increase the movement space of the robot arm.

Moreover, in some studies, the utilization of grippers has been proposed for grasping the steering wheel and controlling it at all times. The robot grabs the center of a steering wheel with one end-effector [13]. However, the size and shape of the steering wheel center vary from one vehicle to another. In addition, there are also some studies on the use of dual arms to control the steering wheel. An autonomous driving project using a musculoskeletal humanoid with both arms is presented [14–16]. In this study, a push–pull steering method is used, and the turning velocity was approximately 180^∘^ in 70 s. In addition to driving vehicles, similar research has been conducted for automatically turning circular valves. An aircraft with a dual arm is presented for valve turning [17]. In [18], a semi-autonomous control framework is proposed and experimentally validated by valve turning. In these methods, both arms are used to rotate so that the forces are symmetrical and increase stability. However, when turning the steering wheel with grippers, frequent gripping and releasing wastes time and results in slow rotation. Because the end-effector is required to grip at the same speed as the steering wheel, after releasing, it pulls outward and moves to the next gripping position.

In this paper, we aim to make the robot able to maneuver the vehicle agilely in a narrow cab. Inspired by human driving strategies, we quantitatively analyze the motion data of human-driven vehicles from 3 aspects, the motion range of joint combinations (RJC), the motion region of shoulder (RS), and the velocity of manipulation (VM). In the joint performance configuration, it is always the case that the performance of adjacent joints is sacrificed in order to increase the range of motion of some joints. Therefore, RJC replaces the range of motion of each joint as an indicator of the driving strategy’s demand for joint range of motion in narrow space. Then, RS describes the translation of the shoulder joint using the minimum bounding sphere. It demonstrates the involvement of the shoulder joint in the driving strategy. Since the design of the shoulder translation joint of the humanoid robot is difficult, the greater the participation of the translation joint in turning motion, the less applicable the strategy is to the robot. In addition, we use the VM metric to reflect the effect of the gripper on the speed of the rotating steering wheel. According to 3 analyses, a friction-driven driving strategy is proposed to ensure that the robot quickly manipulates the steering wheel in a narrow space. During driving, the robot needs to use friction to overcome the steering wheel resistance torque. An operating force model of the steering wheel is designed by robot driving data, accurately describing the relationship between the steering wheel motion state and the resistance torque. Notably, applying constant pressure to the steering wheel from the end effector completes the maneuver. However, this can deform the steering wheel and cause damage. Then, a quadratic programming (QP)-based control framework is proposed to control the end-effector position and the contact wrench applied to the steering wheel. Finally, an experiment is conducted to demonstrate the effectiveness of the proposed method, where a humanoid robot drives a vehicle through a chicane. The main contributions of this paper are summarized as follows.

1. Human driving strategies are quantitatively analyzed from 3 aspects, RJC, RS, and VM. A friction-driven driving strategy is proposed to ensure that the robot quickly manipulates the steering wheel in a narrow space.

2. An operating force model of a steering wheel is proposed to describe the relationship between the steering wheel motion state and the resistance torque for outputting an accurate interaction target wrench.

3. Using the proposed strategy in this paper, an obstacle avoidance scenario is carried out. Moreover, the turning speed reaches 3.14 rad/s (the fastest in research as far as we know).

The rest of this paper is organized as follows. In the “Steering strategy” section, we quantitatively analyze the advantages and disadvantages of 3 different driving strategies, and propose a friction-driven strategy based on the comparative results. In the “Steering Wheel Modeling” section, the relationship between the rotation angle and resistance torque is proposed. The QP-based control framework considering the resistance torque of turning is presented in the “QP-Based Turning Motion Control Framework” section.

The results of the experiment using the proposed method are presented in the “Experiment and Discussion” section. Finally, the conclusions drawn from this study are presented in the “Conclusion” section.

## Steering Strategy

The steering wheel and associated mechanisms have changed dramatically over the years. Thus, recommendations relative to hand position on the steering wheel have become more flexible due to these changes. There are three driver steering strategies that can be used to provide smooth and continuous steering control. We describe these methods as follows, which are recommended by the National Highway Traffic Safety Administration (NHTSA) [19], using the example of turning the steering wheel counterclockwise.

**Fig. 1.**
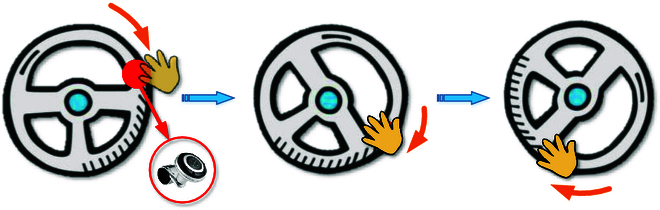
The steering wheel assistive power booster is fixed on wheel represented by a red circle. The hand grabs the booster and rotates the steering wheel clockwise.

1. “Hand-to-Hand” (HtH): First, the left hand grasps the wheel between 7 and 8 o’clock, and the right hand between 4 and 5 o’clock. Second, the left hand pushes the wheel up and the opposite hand slides up, grasps the wheel, and pulls down to turn. Third, while the pulling hand moves down, the hand that initially pushed up slides back toward its original position to make adjustments as needed.

2. “Hand-over-Hand” (HoH): First, the left hand grasps the steering wheel between 8 and 9 o’clock, and the right hand between 3 and 4 o’clock. Second, the right hand pushes up upper right corner of the steering wheel. Third, the opposite hand reaches across the other arm, grasps the wheel, and pulls the wheel up, over, and down as appropriate.

3. “One Hand" (OH): The right hand pushes the wheel using friction force or grasping a steering wheel assistive power booster as shown in Fig. 1. The position of the hand relative to the world coordinate follows the rotation of the steering wheel, while the posture of the hand does not change.

**Fig. 2.**
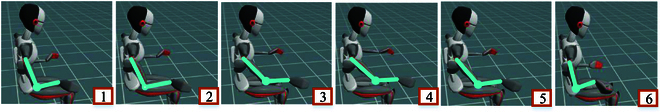
Snapshot of turning using HtH steering.

**Table 1.** Performance of Xsens MVN Link

Static accuracy (roll/pitch)	0.2 deg
Static accuracy (heading)	0.5 deg
Dynamic accuracy	1 deg RMS
Accelerometer range	±16 g
Gyroscope range	±2000 deg/s

For humans, all 3 strategies mentioned above allow for stable and quick manipulation of the steering wheel. However, to determine which strategy is more suitable for humanoid robots, a more detailed analysis of human steering motion is necessary. The data collection phase for human subjects involved the utilization of Xsens’ MVN Link system. This system is a 3-dimensional human motion capture system based on inertial measurement units (IMUs), which comprise multiple IMU sensors including accelerometers, gyroscopes, magnetometers, and other sensors to measure acceleration, angular velocity, orientation, and other body-related information. These sensors are integrated into a motion capture suit worn by the subjects.

The MVN Link system incorporates a biomechanical model that utilizes fixed IMU sensors positioned at key points on the human body, such as the head, arms, trunk, and legs, to capture motion data, the performance of which is shown in Table 1. The sensor data are transmitted wirelessly or via wired connections to a computer, where the collected data are fused to obtain accurate information regarding the pose, position, and velocity of the human body. This enables real-time capture of human motion data, which can be applied in various fields including motion analysis, kinematic studies, motion training, virtual reality, gaming, and more. In this study, the MVN Link motion capture system was employed to record the upper body motion of individuals while using the 3 different steering strategies. Four drivers sat in the cab one after another, start the vehicle, but do not move forward. Each driver turns the steering wheel one turn using 3 different driving strategies. We select the sample with the smallest combined joint motion range from the 4 drivers.

The HtH strategy is used to turn the steering wheel, as shown in Fig. 2. Since there is no sensor to detect finger movements, the fingers and palm are treated as rigid bodies. Figure 2 (1 to 3) demonstrates the simultaneous forward extension of both arms, while Fig. 2 (3 to 6) depicts the simultaneous backward contraction of both arms. Because the hand is holding the steering wheel, the contact zone between the hand and the steering wheel remains unchanged.

**Fig. 3.**
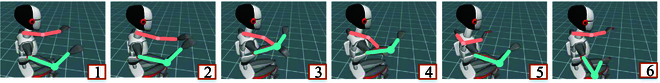
Snapshot of turning using HoH steering.

The HoH strategy is utilized for steering, as shown in Fig. 3, where the left and right arms are depicted in red and blue, respectively. In Fig.3 (1 and 2), it can be observed that the left arm (red link) extends outward, rotating the steering wheel clockwise, while the right arm remains suspended in the air. In Fig. 3 (3), the motion snapshot captures the moment when the hands exchange their positions. As the left hand moves to the 1 to 2 o’clock position, the right hand reaches the 11 o’clock position. Both hands then grasp the steering wheel simultaneously, resulting in the closest possible distance between the forearms.

**Fig. 4.**
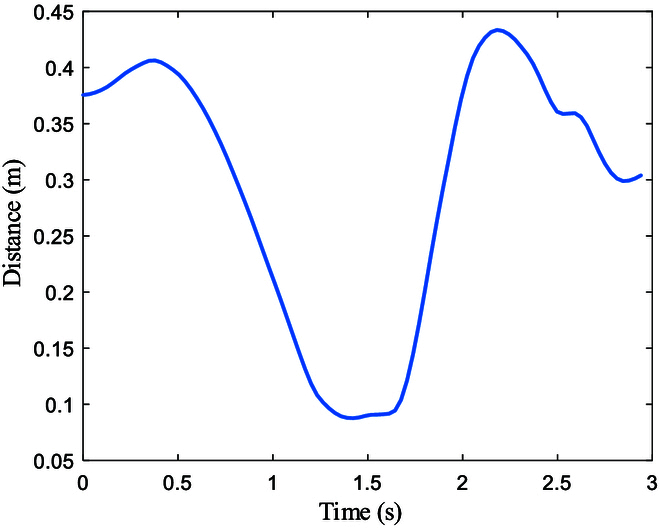
The minimum distance between the forearms with HoH.

The forearm is represented by the lines connecting the elbow joint and wrist joint. The minimum distance between the 2 forearms is 8.76 cm as shown in Fig. 4.

Typically, the average male wrist diameter is 6 to 7 cm, resulting in a minimum distance of 2 to 3 cm between the outer contours of the forearms. For the human body, even if this distance is reduced to zero, it is still considered safe due to the flexibility and elasticity of the muscles and skin tissues.

However, when a humanoid robot assumes a crossed-arm posture similar to humans, the arm closest to the steering wheel needs to be released and retracted, requiring a significant amount of space. Moreover, the gripper and electronic components of the wrist joint are situated inside the forearm, and the mechanical structure must ensure a sufficient range of motion and dynamic performance. This presents a challenge in designing slender forearms that closely resemble the size of a human arm. Additionally, even slight collisions with rigid metal structures can increase the risk of joint damage for the robot.

**Fig. 5.**
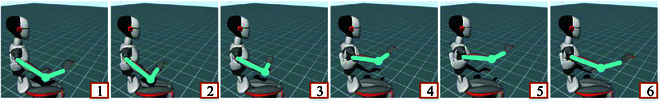
Snapshot of turning using OH steering.

The OH strategy is used to turn the steering wheel, as shown in Fig. 5. The steering wheel is rotated by the frictional force applied by the hand. The right hand is always pressing on the top of the steering wheel, with the back of the hand facing upward in the motion snapshot. The purple curve shown in Fig. 5, representing $\theta_{\text{wrist}}^{\mathrm{yaw}}$, also confirms the aforementioned situation without significant changes.

Regarding the OH strategy, there are 2 methods employed to manipulate the steering wheel. The first method involves utilizing frictional force between the palm and the steering wheel, while the second method entails using a steering wheel assistive booster mounted on the steering wheel. In the latter method, the palm grasps the booster and exerts pressure to rotate the steering wheel. Of course, humans possess the capability to quickly and effortlessly master both methods.

### Motion range of joint combinations

To facilitate a visual comparison of the range of motion needed for each joint under the 3 different strategies, a summarized representation is provided in Table 2. The minimum range of motion required for each joint is highlighted in bold. Generally, the HoH strategy necessitates the largest range of motion.

To further compare the range of motion required across the various strategies, 7 degrees of freedom (DoFs) are divided into 3 combinations: the shoulder, elbow, and wrist. These 3 combinations exhibit a high level of integration in terms of mechanical design, with electronic components often placed on the structural components between the joints to reduce joint volume. Typically, the shoulder joints ($\theta_{\text{shoulder}}^{\text{pitch}}$ and $\theta_{\text{shoulder}}^{\mathrm{yaw}}$) can achieve a range of motion of 360 degrees. However, the elbow and wrist joints face limitations due to the limited space within the forearm, as well as the incorporation of force sensors and actuators (grippers) at the end of the arm. Consequently, it becomes challenging to simultaneously increase the range of motion for all 4 DoFs in the elbow and wrist. In this study, the range of motion requirements for similar positions of the DoF are combined, and the joint combinations are compared. The comparison of the motion range of joint combinations is presented in Table 3.

**Table 2.** Joint angle of human

	HtH			HoH			OH		
Joint	Max	Min	Range	Max	Min	Range	Max	Min	Range
$\theta_{\text{shoulder}}^{\text{roll}}$	35.76^∘^	4.15^∘^	**31.01** ^∘^	37.41^∘^	5.82^∘^	31.59^∘^	45.46^∘^	9.78^∘^	35.68^∘^
$\theta_{\text{shoulder}}^{\mathrm{yaw}}$	36.32^∘^	4.02^∘^	**32.30** ^∘^	63.48^∘^	0.89^∘^	62.59^∘^	64.77^∘^	10.09^∘^	54.68^∘^
$\theta_{\text{shoulder}}^{\text{pitch}}$	75.19^∘^	19.45^∘^	**55.74** ^∘^	86.22^∘^	23.25^∘^	62.97^∘^	80.77^∘^	22.33^∘^	58.43^∘^
$\theta_{\text{elbow}}^{\mathrm{yaw}}$	89.65^∘^	49.19^∘^	40.46^∘^	138.84^∘^	52.63^∘^	86.21^∘^	119.51^∘^	96.08^∘^	**23.42** ^∘^
$\theta_{\text{elbow}}^{\text{pitch}}$	97.21^∘^	29.48^∘^	**67.73** ^∘^	108.68^∘^	19.25^∘^	89.42^∘^	96.07^∘^	22.27^∘^	73.79^∘^
$\theta_{\text{wrist}}^{\text{roll}}$	3.39^∘^	−21.52^∘^	**24.91** ^∘^	19.04^∘^	−21.07^∘^	40.11^∘^	15.32^∘^	−15.46^∘^	30.79^∘^
$\theta_{\text{wrist}}^{\text{pitch}}$	1.22^∘^	−61.33^∘^	62.55^∘^	−0.27^∘^	−36.94^∘^	36.68^∘^	−6.50^∘^	−33.17^∘^	**26.67** ^∘^

**Fig. 6.**
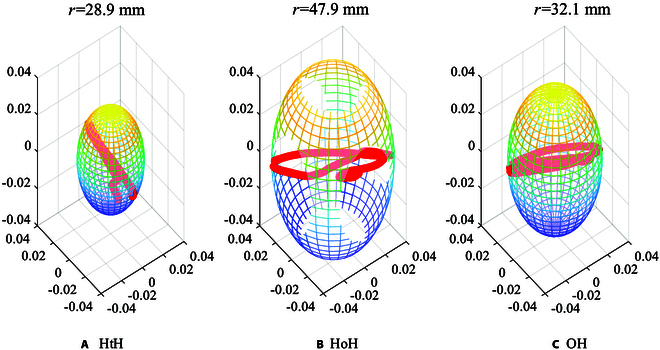
The minimum bounding sphere of the trajectory of the shoulder in space. *r* is the radius of the bounding spheres.

**Fig. 7.**
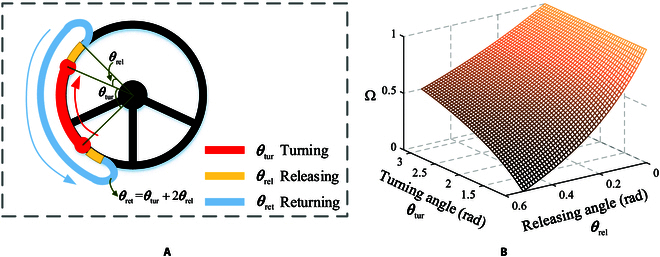
(A) Schematic diagram of the gripping and releasing of the gripper. (B) Relationship between *θ*_tur_ and *θ*_ret_.

**Fig. 8.**
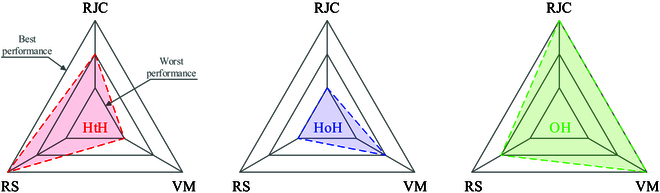
The performance of the 3 driving strategies in 3 aspects: motion range of joint combinations (RJC), motion region of shoulder (RS), and velocity of manipulation (VM).

**Fig. 9.**
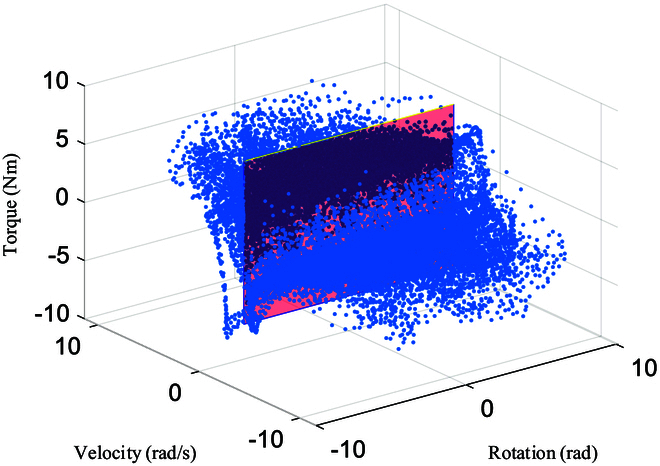
The relationship between the resistance torque *τ_r_* of turning the steering wheel and its rotation state $\left[\theta ,\overset{\cdot }{\theta }\right]$.

**Fig. 10.**
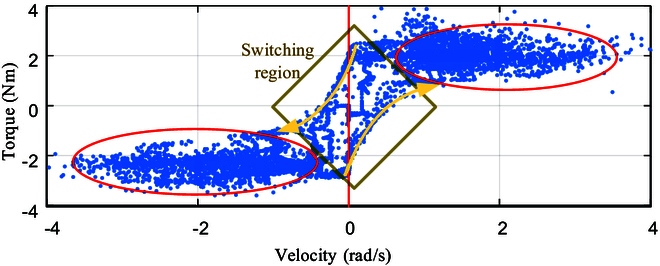
The relationship between the resistance torque *τ_r_* of turning the steering wheel and its rotation velocity $\overset{\cdot }{\theta }$. The blue points are denoted as the sample *X* ∈ *ℝ*^*N* × 2^. The yellow region in the illustration represents the alternating positive and negative directions of rotation. The 2 yellow arrows highlight the fact that the magnitude of torque is dependent on the direction of velocity. The red region demonstrates that when the direction of motion remains constant, there is a relatively minimal change in torque.

**Fig. 11.**
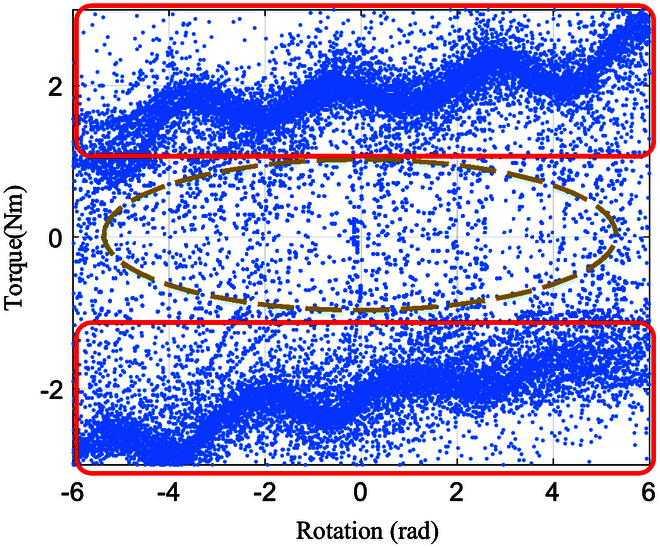
The relationship between the resistance torque *τ_r_* of turning the steering wheel and its rotation velocity.

**Fig. 12.**
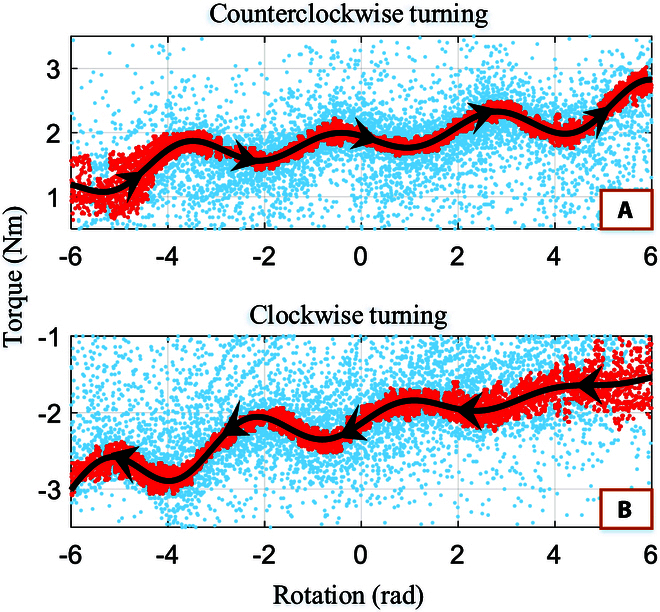
The relationship between *τ_r_* and *θ*. The red points are denoted as the data $\hat{X}$ after rejections. The black lines with arrows represent the function *f_l_*(*θ*). The blue points are denoted as sample *X* ∈ *ℝ*^*N* × 2^. (A) As the steering wheel is turned counterclockwise, the torque changes in the direction of the black line arrow as the angle increases. (B) As the steering wheel is turned clockwise, the torque changes in the direction of the black line arrow as the angle decreases.

**Fig. 13.**
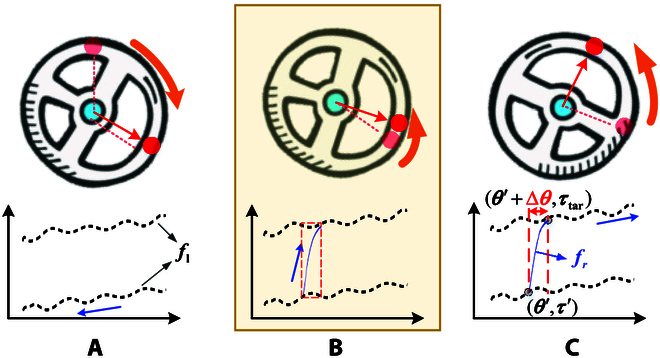
(A) When the steering wheel moves in one direction, such as clockwise, the resistance torque *τ_r_* = *f_l_*(*θ*) is represented as a black dashed line, similar to the black lines with arrows in Fig. 12 (B) The resistance torque changes from *τ*′ to *τ*_tar_. (C) When moving counterclockwise, *τ_r_* = *f_l_*(*θ*).

**Fig. 14.**
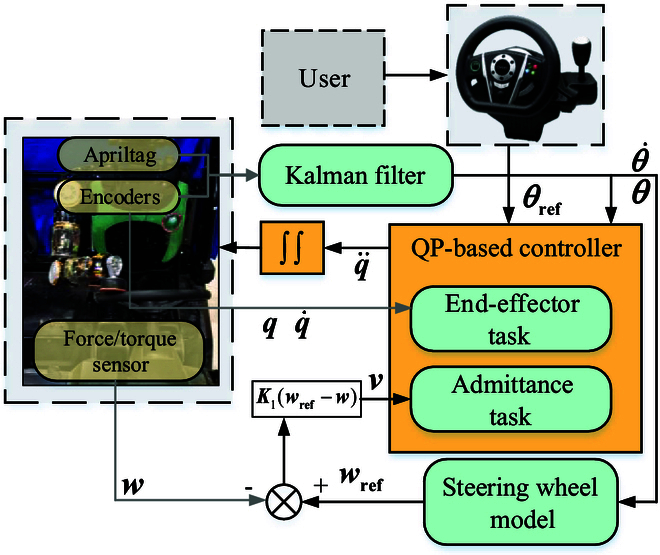
The framework of the QP-based controller. *θ*_ref_ is the target angle of the steering wheel, which is given by the user. In this paper, we use sim racing wheels (Thrustmaster) to output *θ*_ref_ with ROS communication. The steering wheel model is described in the “Steering Wheel Modeling” section, in which the resistance torque *τ_r_* is obtained by *f_r_*(*θ*) or *f_l_*(*θ*) depending on the turning state $\left[\theta ,\overset{\cdot }{\theta }\right]$. The controller contains 2 parts, an end-effector task and an admittance task. The end-effector task is aimed at position and posture tracking of a 1-DoF passive friction flat, admittance task at wrench tracking.

**Fig. 15.**
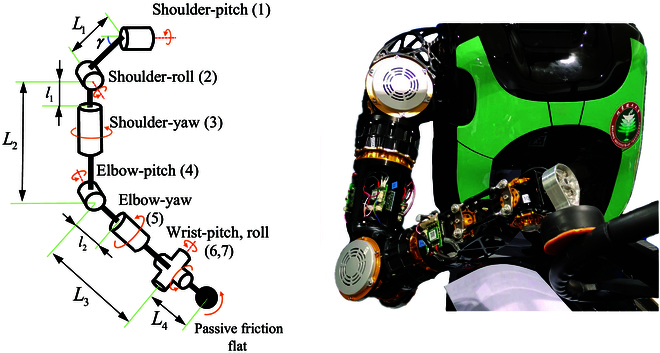
Schematic of the actual arm.

**Fig. 16.**
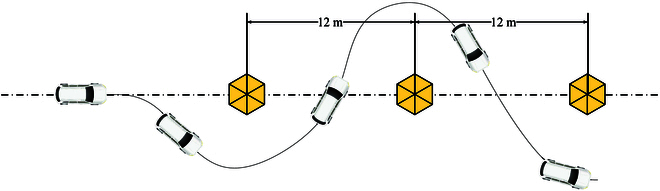
The trajectory of the vehicle. The yellow hexagons indicate the obstacles.

**Fig. 17.**
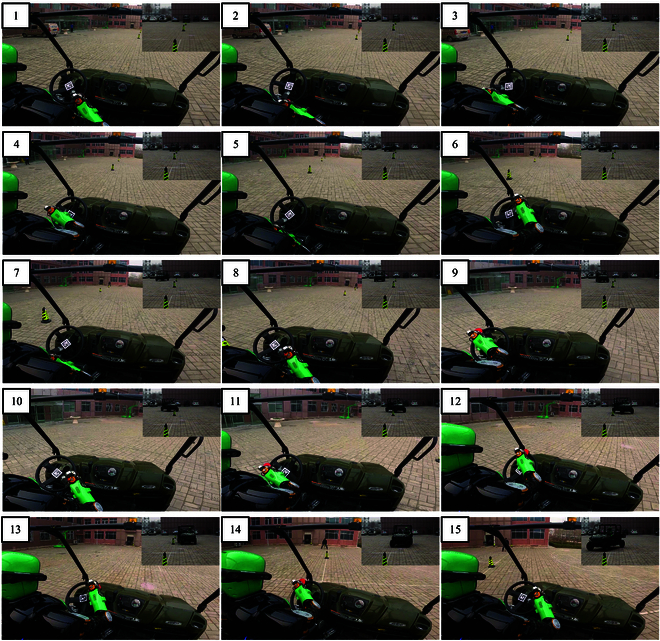
Snapshots of the humanoid robot driving at a chicane.

**Fig. 18.**
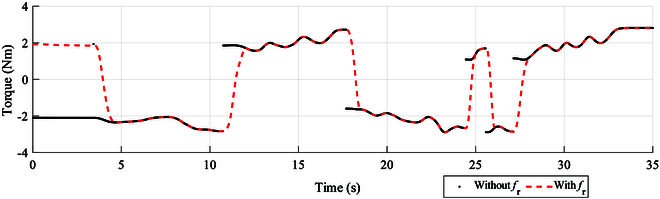
The desire resistance torque is generated by the proposed method described in the “Steering Wheel Modeling” section. The red dashed lines indicate that the resistance torque is generated by *f_l_* and *f_r_*, and the black dots indicate that the resistance torque is generated by only *f_l_*.

**Fig. 19.**
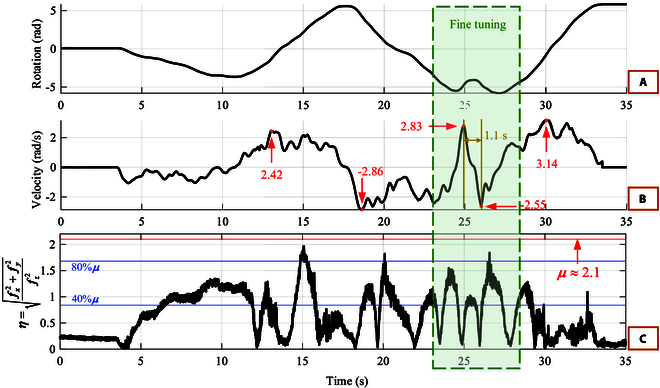
The states of passive friction flat turning the steering wheel when the humanoid robot is driving in a chicane. The green box shows that the robot is tuning the steering wheel, i.e., changing the direction of rotation quickly and frequently over a short period of time. (A) The rotation angle of the steering wheel. (B) The angular velocity of the steering wheel. Some points with high values of the rotation velocity are marked in red. The maximum value reaches 3.14 rad/s. (C) *η* is the ratio of friction force (*f_x_* and *f_y_*) to the vertical pressure (*f_z_*). The red line indicates the friction coefficient of table tennis rubber attached to the passive flat [26]. The 2 blue lines indicate 80 % *μ* and 40 % *μ*.

**Table 3.** Motion range of joint combinations

Joint combinations	HtH	HoH	OH
Elbow	108.19^∘^	175.63^∘^	**97.21** ^∘^
Wrist	87.46^∘^	76.79^∘^	**57.46** ^∘^
Total	195.65^∘^	252.42^∘^	**154.67** ^∘^

**Table 4.** Performance comparison

	HtH	HoH	OH
Joint	Value Rate Score	Value Rate Score	Value Rate Score
RJC	195.65^∘^ 22.49% Fair	252.42^∘^ 0% Bad	154.67^∘^ 38.73% **Good**
RS	28.9 mm 39.67% **Good**	47.9 mm 0% Bad	32.1 mm 32.99% Fair
VM	0.68 rad/s 0% Bad	0.77 rad/s 33.82% Fair	1 rad/s 47.06% **Good**

**Table 5.** Some parameters of the arm

-	*γ*	*L*_1_	*L*_2_	*L*_3_	*L*_4_
Value	0.785 rad	0.100 m	0.294 m	0.292 m	0.060 m
Joint	Torque (N.m)		Speed (rad/s)	Motion boundary (rad)	
Shoulder-pitch	115		7.32	−3.14 to 3.14	
Shoulder-roll	184		4.58	−1.05 to 1.57	
Shoulder-yaw	115		7.32	−3.14 to 3.14	
Elbow-pitch	100		5.76	−1.05 to 1.57	
Elbow-yaw	50		11.52	−3.14 to 3.14	
Wrist-pitch	20		18.84	−1.05 to 1.05	
Wrist-roll	10		25.12	−1.31 to 1.05	

In Table 3, it is noted that the elbow joint has a large range of motion under the HoH strategy, while the wrist joint has a relatively larger range of motion under both the HoH and HtH strategies. This is because the palm needs to maintain the same position and posture as it contacts the steering wheel in the 2 strategies. Compared to the HoH strategy, the total motion range of joint combinations under the HtH and OH strategies reduces the range of motion by 22.49% and 38.73%, respectively, as calculated by Eq. 1.

$$\eta =\frac{\sum_{i=1}^{n}‍\left(R_{1}^{i}-R_{2}^{i}\right)}{\sum_{i=1}^{n}‍R_{1}^{i}}$$ (1)

### Motion region of shoulder

During the motion of turning the steering wheel, the shoulder joint undergoes a slight anterior–posterior displacement to achieve a more comfortable posture. The freedom of anterior–posterior movement effectively addresses the issue of limited workspace caused by insufficient arm length. It is worth noting that increasing the arm length expands the workspace, but when manipulating in close proximity to the trunk, larger joint ranges are required to facilitate the folding motion between the upper and lower arms. The human shoulder joint is a complex system comprised of several interrelated components, including the sternoclavicular joint, acromioclavicular joint, glenohumeral joint, and scapulothoracic joint. These joints are coupled together, so it is difficult to analyze them independently as in the case of arm rotational joints. Therefore, the utilization of a minimum bounding sphere is proposed to describe the motion region of the shoulder [20].

As shown in Fig. 6, the motion region of the shoulder with the 3 strategies can be assessed using the minimum bounding sphere. Although the largest sphere radius appears relatively small in the graph, measuring 47.9 mm, achieving linear motion of the shoulder joint is challenging for humanoid robots. The HtH strategy exhibits the smallest minimum bounding sphere radius, indicating the least required motion region for the shoulder. The hand does not need to reach the farthest end of the steering wheel, such as between the 12 o’clock and 2 o’clock positions, while the other 2 strategies require hand contact with the far end of the wheel. Conversely, the HoH strategy demonstrates the largest minimum sphere radius, indicating the largest required motion region. This is because the hand not only needs to reach the far end of the steering wheel but also performs grasping actions, further increasing the motion region requirements for the shoulder.

### Velocity of manipulation

For both the HtH and HoH strategies mentioned above, the hand needs to repeatedly grasp and release the steering wheel. Mechanical grippers are far less agile than human hands, and their gripping action involves 2 steps: the fingers move to a width equal to that of the object and then apply the preloading force. Before gripping the object, the width between the fingers needs to be greater than the width of the object. Upon contact, the width between the fingers and width of the object is roughly the same, and it is necessary to ensure that the relative velocity between the gripper and the object is zero. Due to uncertainties in the object size, pose, and modeling, it is difficult to determine the state of the object at the moment of gripping. Therefore, the gripper needs to maintain the movement speed of the object for a short time until the end of the grasp. For example, with the HtH strategy, the left hand manipulates the steering wheel from 8 o’clock to 10 o’clock, i.e., gripping at 8 o’clock and releasing at 10 o’clock. To ensure that the griper can be completed at 8 o’clock, the griper needs to be accelerated to target speed before 8 o’clock (e.g., 7 o’clock) and then the finger width (of the mechanical gripper) decreases rapidly to ensure preloading at 8 o’clock.

To quantitatively analyze the effect of the gripping and releasing motions of the gripper on the manipulation velocity with different strategies, we divide the gripper trajectory during the manipulation of the steering wheel into 3 parts, turning, releasing, and returning as shown in Fig. 7. We transform these trajectories into steering wheel angles (*θ*_tur_, *θ*_rel_, *θ*_ret_) for easy description. Therefore, the time of turning and releasing can be written as

$$\left\{\begin{array}{l} t_{\text{tur}}=\frac{\theta_{\text{tur}}}{\omega_{\text{tur}}}\\ t_{\text{rel}}=\frac{\theta_{\text{rel}}}{\omega_{\text{tur}}} \end{array}\right.$$ (2)

and the time of returning is

$$t_{\text{tur}}=t_{\text{ret}}+2t_{\text{rel}}$$ (3)

Then, the relationship between *ω*_tur_ and *ω*_ret_ can be written as

$$\Omega =\frac{\omega_{\text{tur}}}{\omega_{\text{ret}}}=\frac{\theta_{\text{tur}}-2\theta_{\text{rel}}}{\theta_{\text{tur}}+2\theta_{\text{rel}}}$$ (4)

Since Ω is always less than 1, *ω*_tur_ is always less than *ω*_ret_. We assume that *ω*_ret_ is the maximum velocity of the arm end-effector. In fact, the maximum velocity of the end-effector is not a constant value, but a variable related to the posture. However, for the convenience of comparison, the maximum velocity is assumed to be a constant value. The changing trend of Ω is shown in Fig. 7B. Both a small *θ*_tur_ and a large *θ*_rel_ result in a reduction in the velocity of manipulation.

### Summary of analysis

Because the OH strategy has no gripping and releasing motion, it can maximize the performance of the robot arm, i.e., the maximum velocity of rotation with external wrench is approximately equal to the maximum velocity of manipulation the steering wheel. The frequent gripping and releasing, acceleration, and deceleration of the end-effector will make the velocity of the robot arm in the return trip much larger than the velocity of rotating steering wheel as described in the “Velocity of manipulation” section. However, the posture of the palm relative to the contact point on the steering wheel will change with rotation, requiring the end effector of the arm to maintain control of the steering wheel while experiencing relative sliding, demanding precise control of frictional forces. As for the 2-handed strategy (HtH and HoH), a major advantage is the control stability since the steering wheel is constantly held by the hands, making it easy to prevent any sliding between the end effector and the steering wheel. We use radar figures to visualize the above 3 aspects as shown Fig. 8. The OH strategy is best at RJC and VM.

Therefore, we propose an OH-based friction-driven strategy that can provide strong support for agile manipulation based on the analysis of Fig. 8. Detailed data are shown in Table 4. Rate represents the percentage of improvement compared to the worst. The scores are divided into 3 categories: good, fair, and bad. The OH strategy has the highest score. For VM, we set 1 rad/s for OH strategy and *θ*_rel_ = 0.2 rad. Then, the VM values of HtH and HoH are obtained by Eq. 4.

## Steering Wheel Modeling

Electric power steering (EPS), including motors, electronic control unit (ECU), torque sensors, and encoders, are commonly equipped on vehicles. It estimates the input state of steering wheel (rotation position *θ*, speed $\overset{\cdot }{\theta }$) and the velocity of the vehicle and controls the output torque of the motor to reduce the turning resistance. The relationship between the inputs and outputs of the EPS is a black box for users. Besides, with complex mechanical transmission systems, including universal joints, rack, and pinion, it is difficult to build an accurate mathematical model to describe the entire drive process from the steering wheel to the tires. Meanwhile, our strategy is to use friction to turn the steering wheel. Once the friction cannot overcome the resistance, the end effector will slip on the steering wheel and hence lose control of it. Therefore, an accurate resistance reference is important for control using friction.

To model the steering wheel, namely, to obtain the relationship between the wheel state $\left[\theta ,\overset{\cdot }{\theta }\right]$ and the resistance torque, the robot obtains this information from a force/torque sensor and encoders in joints. In addition, because hand slipping is inevitable, a long experiment can lead to *θ* drift if the end-effector movement is considered steering wheel rotation. Therefore, we obtain *θ* as the measurement by AprilTag [21], in which quick response (QR) codes are attached on the wheel and a camera is installed on the robot head. Then, an accuracy state is obtained by fusing data through forward kinematic and visual data through a Kalman filter (KF).

Using the steering wheel state (*θ* and $\overset{\cdot }{\theta }$) obtained from the previous KF output and combining it with the force/torque sensor information during rotation, the relationship between the steering wheel resistance torque and *θ* and $\overset{\cdot }{\theta }$ can be obtained, as shown in Fig. 9.

In Fig. 9, the blue data points represent the relationship between the torque applied to the steering wheel by the robot and the steering wheel rotation angle and velocity during each control loop. The transparent red plane represents $\overset{\cdot }{\theta }=0$. The steering wheel rotation angle and velocity are obtained by KF. The force applied to the steering wheel by the robot is obtained using a force/torque sensor, and the coordinate transformation is as follows:

$$\left\{\begin{array}{c} F_{\text{steering}}=R_{\text{steering}}^{\text{sensor}}F_{\text{sensor}}\\ \tau_{\text{steering}}=R_{\text{steering}}^{\text{sensor}}\tau_{\text{sensor}}+\left(r_{\text{steering}}-r_{\text{sensor}}\right)\times F_{\text{sensor}} \end{array}\right.$$ (5)

where *F*_steering_ = [*f_x_*, *f_y_*, *f_z_*]^⊤^ and *τ*_steering_ = [*τ_x_*, *τ_y_*, *τ_z_*]^⊤^ represent the force applied to the steering wheel by the robot. *F*_sensor_ and *τ*_sensor_ represent the information measured by the force/torque sensor at the robot end-effector. *r*_steering_ and *r*_sensor_ represent the positions of the end-effector contact point and force/torque sensor in the world coordinate system. $R_{\text{steering}}^{\text{sensor}}\in {\mathbb{R}}^{3\times 3}$ denotes the rotation matrix that transforms coordinates from the force/torque sensor frame *R*_sensor_ to the frame of the contact point *R*_steering_, that is, $R_{\text{steering}}^{\text{sensor}}=R_{\text{sensor}}^{⊤}R_{\text{steering}}$.

Due to the lack of accurate parameters for the steering wheel model, it is difficult to prevent slipping during the rotation of the steering wheel by the humanoid robot. Excluding slipping data can make the data more realistic. In this paper, we assume that the static friction force is equal to the sliding friction force, as follows:

$$\sqrt{f_{x}^{2}+f_{y}^{2}}\geq \mu f_{z}\cdot \eta_{f}$$ (6)

where *η_f_* represents the exclusion coefficient, which is set by the user. Using Eq. 6, data with frictional forces close to *μf_z_* are excluded to obtain valid data. We analyze the relationship between the steering wheel resistance and the rotational speed and position separately.

The blue dots in Fig. 10 represent the extracted samples *X* ∈ *ℝ*^*N* × 2^ for each control cycle. The yellow diamond-shaped region represents the switch between positive and negative rotation directions. The 2 yellow arrows describe the boundary between the relationship of torque and rotation velocity in the switching region. The red region represents the same direction region, where the distribution of blue data points remains the same when the rotational direction remains unchanged. The data are divided into 2 regions: the switching region and the same direction region.

1. Same direction region: The red elliptical shape describes the relationship between the torque and rotation velocity when the rotation direction does not change. When the velocity is positive, the torque is positive; when the velocity is negative, the torque is negative. The torque does not significantly change with the absolute value of the rotation velocity in the same rotational direction.

2. Switching region: The yellow diamond-shaped box describes the switching region between positive and negative rotation directions. Due to the angle limit of the steering wheel, the robot cannot continue to rotate in the same direction. When the robot switches the rotation direction, the data points fall into the switching region. The yellow curved arrow illustrates the change in the torque when the robot quickly changes direction, that is, when the rotational velocity changes from $\overset{\cdot }{\theta }=0$ to $|\overset{\cdot }{\theta }|>0$. When the velocity changes slowly, the data points fall between the 2 yellow lines.

As shown in Fig. 10, after eliminating points for block slippage, when the steering wheel is moving in the same direction, there is no significant change in *τ_r_* as $\overset{\cdot }{\theta }$ increases. As $|\overset{\cdot }{\theta }|<4$, there is no significant correlation between both. The direction of motion will change during continuous rotation of the steering wheel, i.e., positive and negative switching of the velocity, causing *τ_r_* to change rapidly in the yellow region.

The relationship between the torque *τ_r_* and the rotation angle is shown in Fig. 11. Based on the distribution of the blue data points, the steering wheel rotation is classified into 3 categories, represented by 2 red boxes and a yellow oval box. From the analysis of Fig. 10, the direction of the torque is positively correlated with the direction of motion (i.e., the sign of the rotation velocity). Thus, the 2 red boxes represent the relationship between the torque *τ_r_* and the rotation velocity *θ* when rotating in the same direction, while the yellow oval box represents the relationship when the robot is capable of reversing its rotation direction. Since the data for the reverse rotation are significantly less than those for same-direction rotation, we use the Grubbs test [*22*] to remove any outliers to investigate the relationship between torque and rotation angle during same-direction rotation.

In this paper, the data points *X* in the red boxes in Fig. 11 are assumed to follow a normal distribution with a mean of *μ* and a standard deviation of *σ*. The corresponding standardized normal distribution random variable is denoted as $Z=\frac{X-\mu }{\sigma }$. The sample mean $\bar{X}$ is used to estimate the population mean *μ*, while the sample standard deviation *S* is used to estimate the population standard deviation *σ*. A similar standardization process is then performed to obtain the corresponding statistics of the standard normal distribution. The Grubbs test statistic is then calculated by plugging in the sample mean $\bar{X}$ and sample standard deviation *S* of the steering wheel rotation.

$$G=\frac{\overset{n}{\underset{i=1}{\text{max}}}|X_{i}-\bar{X}|}{S}$$ (7)

where

$$S=\sqrt{\frac{1}{n-1}\sum_{i=1}^{n}‍{\left(X_{i}-\bar{X}\right)}^{2}}$$ (8)

According to the central limit theorem, when the sample size is sufficiently large, the distribution of the sample mean $\bar{X}$ is approximately normal. In this case, we can use the sample mean $\bar{X}$ to replace the population mean *μ*, use *S* to replace the population standard deviation *σ*, and then standardize them, obtaining the following equation:

$$Z_{i}=\frac{X_{i}-\bar{X}}{S}$$ (9)

where *Z_i_* represents the standardized value of the *i*th data point *X_i_*. Since each data point can be standardized, we can find the data point with the largest standardized value

$$G=\max_{i=1}^{n}|Z_{i}|$$ (10)

Then, we can use the upper quantile of the standard normal distribution, denoted as *Z*_*α*/(2*n*)_, to calculate the critical value of the Grubbs test

$$G_{\alpha }=\frac{\left(n-1\right)/\sqrt{n}\cdot \sqrt{Z_{\alpha /\left(2n\right)}}}{\sqrt{n-2+Z_{\alpha /\left(2n\right)}}}$$ (11)

where *α* represents the significance level and *n* represents the size of the sample. When the sample size is large enough, *Z*_*α*/(2*n*)_ can be replaced by the upper quantile *z*_*α*/2_ of the standard normal distribution, namely:

$$G_{\alpha }\approx \frac{\left(n-1\right)/\sqrt{n}\cdot \sqrt{z_{\alpha /2}}}{\sqrt{n-2+z_{\alpha /2}}}$$ (12)

*z*_*α*/2_ is the upper percentile of the standard normal distribution, which is the point where the upper tail area is *α*/2. The calculation formula is:

$$z_{\alpha /2}=\Phi^{-1}\left(1-\alpha /2\right)$$ (13)

where Φ^−1^ is the inverse function of the cumulative distribution function of the standard normal distribution. In this paper, we set *α* = 0.05, then set *α*/2 = 0.025. The corresponding *z*_0.025_ is

$$z_{\alpha /2}=z_{0.025}=\Phi^{-1}\left(1-0.025\right)=1.96$$ (14)

*G_α_* is a critical value that represents the threshold of the Grubbs test statistic at a given significance level *α* and the number of observed values *n*. If the test statistic exceeds this threshold, the data point can be considered an outlier. Therefore, the condition for removing data points is *G* > *G_α_*. After removal, Eqs. 7 and 12 are recalculated and compared with the critical value until no *G* is larger that *G_α_*. Completion of the rejection makes the relationship between the 2 clearly shown in Fig. 12 as red points.

Then, least squares is used to fit the function of turning without switching direction (from one limitation to another), represented by the black dashed lines.

Above, we obtain the relationship between the reference resistance torque *τ_r_* and the angle of the steering wheel *θ* from one limiting position to another. However, there are many situations in which the turning direction always changes. In this case, the desired resistance torque is required to smoothly transition from a point on the black line to a point on the other black line (in Fig. 12). We set the resistance torque as a kind of sign function as shown in Fig. 13B. When the steering wheel is in a state (*θ*′, *τ*′) and the robot detects a change in the direction of rotation, the torque can be calculated by:

$$f_{r}\left(\theta \right)=\left(\frac{2}{1+e^{a\left(\theta -\theta \prime \right)}}-1\right)\left(\tau_{\mathrm{tar}}-\tau \prime \right)+\tau \prime$$ (15)

where *a* is a negative constant to guarantee *e*^−|*a*Δ*θ*|^ ≈ 0; (*θ*′, *τ*′) denotes the direction starting to change as $\overset{\cdot }{\theta }=0$. Δ*θ* is the angle required to switch from one curve of *f_l_* to another. For example, switching from the curve below to the curve above via *f_r_* is shown in Fig. 13. (*θ* ′  + Δ*θ*, *τ_tar_*) is the intersection of *f_r_* and *f_l_*. After transition, *τ_r_* is calculated by *f_l_*, as shown in Fig. 13C.

## QP-Based Turning Motion Control Framework

We present the whole framework and QP-based controller [24] in this section, as shown in yellow block in Fig. 14. Turning the steering wheel is divided into 2 tasks.

One task is the end-effector task aiming at minimizing the current wheel angle and target. A pose constraint is set between 1-DoF passive friction flat and a frame of the steering wheel so that the flat and wheel move together. The cost function of this task is as follows:

$$\min_{\ddot{q},\tau ,w}\;J_{1}\left(q\right)=\frac{1}{2}{\left\|\ddot{\theta }-{\ddot{\theta }}_{t}\right\|}^{2}$$ (16)

with the residua

$${\ddot{\theta }}_{t}=\theta_{\mathrm{ref}}-P_{1}e-D_{1}\overset{\cdot }{e},\;e=\theta -\theta_{\mathrm{ref}}$$ (17)

where *θ_t_* denotes the end-effector task and ${\ddot{\theta }}_{t}$ denotes the desired task accelerations [23,24]. *τ* is the joint torque. *w* is the contact wrench, including [*f_x_*,*f_y_*,*f_z_*,*τ_x_*,*τ_y_*,*τ_z_*]. *P*_1_ and *D*_1_ denote the task gain, and *θ*_ref_ is a reference of the steering wheel, as shown in Fig. 14.

Another task is the admittance task for tracking reference wrench *w*_ref_ calculated by *τ_r_* obtained in the “Steering Wheel Modeling” section,

$$\begin{array}{c} f_{x}^{\mathrm{ref}}≔\tau_{r}r_{s}\\ f_{z}^{\mathrm{ref}}≔f_{x}^{\mathrm{ref}}/\mu +\delta \end{array}$$ (18)

where *r_s_* is the radius of the steering wheel and *δ* is the value for guaranteeing that *f_x_* set by users is large enough. The others of *w*_ref_ are set to zero. The cost function of this task is written as,

$$\min_{\ddot{q},\tau ,w}J_{2}\left(q\right)={\left\|P_{2}\left(g-g_{d}\right)+D_{2}\left(\overset{\cdot }{g}-{\overset{\cdot }{g}}_{d}\right)+\left(\ddot{g}-{\ddot{g}}_{d}\right)\right\|}^{2}$$ (19)

where *g_d_* =  ∫  ‍ *vdt*, ${\overset{\cdot }{g}}_{d}=\mathrm{vdt}$, and ${\ddot{g}}_{d}=\frac{d}{\mathrm{dt}}v$; *P*_2_ and *D*_2_ denote the admittance gain. For *v*, we have:

$$v=K_{1}\left(w_{\mathrm{ref}}-w\right)$$ (20)

The error between the target wrench and the sensed wrench is converted into the velocity *v* with the diagonal matrix gain *K*_1_. To prevent the end-effector from moving too fast near the contact flat, we add a constraint (*v*_min_, *v*_max_) for *v*. Then, we apply a low-pass filter to prevent shaking of the end-effector.

## Experiment and Discussion

In this section, we present an experiment of a turning steering wheel using a humanoid robot with a 7-DoF arm [25], as shown in Fig. 15. The length of the arm and the performance of the joints are shown in Table 5. The vehicle used in the experiment is the same as that used in the 2015 Darpa robotics challenge.

The turning experiment is carried out using the humanoid robot arm, which has the mechanical structure of the end of the robot arm (Fig. 15) and a 6-axis force/torque sensor (M3714B2, Sunrise Instruments), as shown in Fig. 15. The leg of the humanoid robot is used to press the gas and brake.

As shown in Fig. 16, the humanoid robot drives the vehicle at a chicane (meaning that the vehicle makes an S-curve movement around obstacles). As shown in Fig. 17, a QR code is attached to the center of the steering wheel. The camera installed on the robot head obtains the rotation angle of the QR code (which is also the rotation angle of the steering wheel). The robot is teleoperated by the user. The user observes obstacles around the vehicle via the camera on the robot head and sends commands using the sim racing wheels shown in Fig. 14. As shown in Fig. 18, the black line is generated by the black fitted curve according to *θ*_ref_. The desired resistance torque has significant abrupt changes, which improves with the addition of Eq. 15.

Figure 19A shows the rotation angle of the steering wheel as the vehicle moves to the left and right while moving forward. In Fig. 19B, red points present the high velocity (absolute value > 2) of the steering wheel. These points prove that the robot can turn the steering wheel quickly. At the same time, the vehicle can maintain a forward velocity of about 6 km/h. Figure 19C shows the ratio *η* of the friction force to vertical pressure. If most of the points of the Fig. 19C curve are close to *μ*, there is a risk of passive friction flat slipping at any time. Conversely, if the pressure is a large constant value, *η* approaches 0. A large pressure over a long period of time leads to overheating and damage to the robot joints. By eliminating the start and end stationary phases, 54% *η* (in Fig. 19C) are distributed between 40%*μ* and 80%*μ*. This indicates that our method is able to take full advantage of the frictional properties of the material while ensuring no slippage in the vast majority of cases.

As shown in the green box in Fig. 19, the operator perceives a premature change in direction and therefore fine-tunes the vehicle’s direction of motion. It is common for experienced drivers to do this when a driver realizes that the steering wheel angle does not correspond to the shape of the bend. During the fine-tuning process, the robot changed the steering wheel rotation velocity from 2.83 rad/s to −2.55 rad/s in 1.1 s in Fig. 19B. Besides, the vast majority of *η* are within the margin of safety (less than 80 % *μ*) as shown in Fig. 19C. It illustrates that the robot is able to change the turning velocity quickly to ensure flexibility.

## Conclusion

We quantitatively analyze human driving strategies and propose a friction-driven driving strategy that can ensure that the robot can quickly manipulate the steering wheel in a narrow space. To guarantee that friction can overcome resistance torque, we propose an operating force model describing the relationship between the torque and the state of the steering wheel (*θ* and $\overset{\cdot }{\theta }$). This model is used to determine the accurate wrench that the robot must apply to control the steering wheel. Then, a QP-based control framework is used to track both the position and contact wrench. Finally, we demonstrate the effectiveness of our proposed method through an obstacle avoidance scenario in which a humanoid robot drives an unmodified vehicle through a chicane. Our results show that the maximum rotation velocity can reach 3.14 rad/s, making it an agile solution for steering wheel control.

## Data Availability

The data that support the findings of this study are available from the corresponding author upon reasonable request.
